# The seven constitutive respiratory defense barriers against SARS-CoV-2 infection

**DOI:** 10.1590/0037-8682-0461-2021

**Published:** 2021-12-17

**Authors:** Eduardo Tosta

**Affiliations:** 1 Professor Emeritus, Faculdade de Medicina, Universidade de Brasília, Brasília, DF , Brasil.

**Keywords:** Respiratory tract microbiota, Defensins and lactoferrin, Surfactant proteins, Epithelial cell interferons, Innate immunity

## Abstract

Before eliciting an adaptive immune response, SARS-CoV-2 must overcome seven constitutive respiratory defense barriers. The first is the mucus covering the respiratory tract’s luminal surface, which entraps inhaled particles, including infectious agents, and eliminates them by mucociliary clearance. The second barrier comprises various components present in the airway lining fluid, the surfactants. Besides providing low surface tension that allows efficient gas exchange at the alveoli, surfactants inhibit the invasion of epithelial cells by respiratory viruses, enhance pathogen uptake by phagocytes, and regulate immune cells’ functions. The respiratory tract microbiota constitutes the third defense barrier against SARS-CoV-2. It activates the innate and adaptive immune cells and elicits anti-infectious molecules such as secretory IgA antibodies, defensins, and interferons. The fourth defense barrier comprises the antimicrobial peptides defensins, and lactoferrin. They show direct antiviral activity, inhibit viral fusion, and modulate the innate and adaptive immune responses. Secretory IgA antibodies, the fifth defense barrier, besides protecting the local microbiota against noxious agents, also inhibit SARS-CoV-2 cell invasion. If the virus overcomes this barrier, it reaches its target, the respiratory epithelial cells. However, these cells also act as a defense barrier, the sixth one, since they hinder the virus’ access to receptors and produce antiviral and immunomodulatory molecules such as interferons, lactoferrin, and defensins. Finally, the sensing of the virus by the cells of innate immunity, the last constitutive defense barrier, elicits a cascade of signals that activate adaptive immune cells and may inhibit the development of productive infection. The subject of the present essay is discussing these mechanisms.

## INTRODUCTION

SARS-CoV-2 needs to bind, enter, and replicate into the respiratory tract’s epithelial cells to originate a productive pulmonary infection. However, to reach these cells, the virus must overcome several constitutive defensive barriers, such as the mucus and mucociliary clearance, surfactant proteins, the respiratory tract microbiota, antimicrobial peptides, and secretory IgA antibodies. Furthermore, the respiratory epithelial cells have mechanisms to counter virus infection, which are amplified by two other defense barriers, the innate and the adaptive immune systems. This review pinpoints how the seven constitutive respiratory defense barriers act to hinder SARS-CoV-2 infection. 

### The 1 ^st^ barrier: Mucus and mucociliary clearance 

The airway mucus constitutes a thin layer of a dense, gel-like material covering the respiratory tract’s luminal surface. The mucus’s primary function is to protect the lungs through the mucociliary clearance of inhaled foreign particles, including infectious agents and noxious pollutants. Mucins comprise the mucus’ major protein component and are present as secreted and cell-associated glycoproteins^1^. The five main mucins, out of the 15 characterized in the human respiratory tract, are distributed in two distinct yet interacting layers. The outer or mucus layer contains two gel-forming mucins (MUC5AC and MUC5B) tightly associated with various defensive molecules. The inner or periciliary liquid layer contains three membrane-tethered mucins (MUC1, MUC4, and MUC16) shed from the apical cell surface^2^. Secreted MUC5AC and MUC5B act as a physical barrier by binding to pathogens, besides performing an immunomodulatory role by capturing, retaining and releasing cytokines and growth factors^1^. The membrane-bound mucins, MUC1, MUC4, and MUC16, form the cell glycocalyx and activate intracellular signal transduction pathways that control epithelial cell shape, differentiation, and proliferation and modulate the inflammatory and immune responses to infectious agents^3^. 

The mucociliary clearance performed by the respiratory epithelium depends on the interactions between mucus and cilia. While the mucus entraps inhaled pathogens and other particulate material, the coordinated beating of cilia sweeps the trapped material away from the lungs toward the pharynx and mouth, where they are swallowed or expectorated. The efficient transport of mucus depends on the ciliary beating rate and the mucus’s hydration, which contributes to its viscoelastic properties. In general, more hydrated mucus is cleared more efficiently from the lungs^4^. During winter, the high transmission of the three major respiratory viruses (influenza viruses, respiratory syncytial virus, and human coronaviruses) may be due to the disruption of mucociliary clearance by the season’s cold and dry air characteristics^5^. A study of the effect of ambient temperature on the ciliary beat frequency of the nasal and tracheal ciliated cells isolated from human subjects showed that mucociliary beating begins to decline as the ambient temperature dips below 20°C and is no longer observed at 5°C^5^. Moreover, the relative humidity (RH) of 10% decreases mucociliary clearance compared to 50% RH, resulting in impaired viral clearance following influenza virus infection of mice^6^ ^,^ ^7^. 

The mucus’ proper hydration maintains the protective effect of the first defense barrier by avoiding the deterioration of the ciliary function by low temperatures and reduced humidity. Air pollutants, mainly smoking, are major disruptors of the mucociliary function. It has been extensively demonstrated that smoking causes its dysfunction and damage, including shortening of airway cilia^8^, besides increasing the expression of ACE2, the cell receptor of SARS-CoV-2^9^. Furthermore, drugs as glucocorticoids^10^ and macrolide antibiotics^11^ inhibit mucin secretion, while others such as anticholinergics, aspirin, anesthetic agents, and benzodiazepines depress the mucociliary transport system^12^ and, hence, disrupt the mucociliary protective barrier. 

### The 2 ^nd^ barrier: Surfactant proteins 

The second barrier that respiratory viruses, such as SARS-CoV-2, must overcome is a variety of soluble inhibitors in the airway lining fluid, collectively known as surfactant proteins or surfactants. Surfactants are complexes of phospholipids with four surfactant-associated proteins (SP-A, SP-B, SP-C, and SP-D), produced mainly by type II alveolar cells. They provide low surface tension to the 100 to 150 m^2^ of alveolar epithelium necessary to allow efficient gas exchange and prevent alveolar collapse and flooding^13^. SP-A and SP-D are family members of immune proteins known as collectins, or collagen-like lectins, which interact with pathogens through their lectin domains and regulate the functions of T lymphocytes, macrophages, dendritic cells, and neutrophils^14^ ^,^ ^15^. Surfactant proteins also inhibit the invasion of epithelial cells by several respiratory viruses^16^. The sharing of 17 pentapeptides of SARS-CoV-2 spike glycoprotein with surfactant molecules^17^ may explain the ability of SP-D to neutralize the virus and enhance its phagocytosis^18^. However, the capacity of SP-D to enhance phagocytosis may lead to macrophage activation, which is involved in the development of the respiratory distress syndrome associated with severe cases of Covid-19^19^. 

The finding that SARS-CoV-2 can downregulate surfactant proteins and their regulators^20^, probably due to the disruption of type II alveolar cells^21^, has posed the possibility of using commercially available surfactants as an adjuvant treatment for Covid-19 pneumonia^21^ ^,^ ^22^. In addition, the demonstration that the lung mechanics in Covid-19 pneumonia resembles respiratory distress syndrome caused by surfactant deficiency^22^ strengthens this claim. 

### The 3 ^rd^ barrier: Respiratory tract microbiota 

The respiratory viruses that survive the inhibitory effects of surfactant proteins must overcome another barrier: the respiratory tract microbiota. The microbiota is a collection of microorganisms (bacteria, viruses, fungi, and archaea) that inhabit the respiratory mucosa, keep a symbiotic relationship with the organism, and play a significant role in shaping the immune system and maintaining homeostasis^23^ ^-^ ^26^. As humans are coevolving with microorganisms since our species’ diversion, the microbiota is considered an evolutionary advantage^27^. Indeed, it has been shown that the respiratory tract microbiota acts as a barrier against the invasion of pathogens, including respiratory viruses, such as influenza virus^28^, respiratory syncytial virus^29^, and SARS-CoV-2^30^. The antivirus environment elicited by the respiratory tract microbiome includes activated innate and adaptive immune cells and anti-infectious molecules such as secretory IgA antibodies, defensins, acetate, and interferons^29^ ^,^ ^31^. 

The association between microbial dysbiosis - disruption of either the composition or the overall number of microbiota components - and increased morbidity and mortality of several respiratory infections validates the importance of the respiratory microbiota for human health^32^. Frequent causes of respiratory tract dysbiosis are antibiotics and intranasal corticosteroids^33^, smoking^34^, and respiratory inflammations, such as asthma and chronic obstructive pulmonary disease^35^. Dysbiosis facilitates SARS-CoV-2 infection, and its causative factors are considered risk factors for severe disease. Moreover, it has been shown that SARS-CoV-2 causes dysbiosis of respiratory microbiota^36^ ^,^ ^37^, which may facilitate the occurrence of complex mixed bacterial and fungal lung infections, a major cause of death of Covid-19 patients^38^. 

The best ways to maintain the respiratory microbiota as a significant defensive barrier are avoiding its disruption by drugs and smoking and strengthening its anti-pathogen capacity by using probiotic microorganisms*.* Clinical and experimental studies have demonstrated that probiotics exert a protective activity against respiratory viruses. Indeed, a Cochrane meta-analysis of 12 randomized control trials including 3,720 adults and children reported a 2-fold lower risk of developing upper respiratory tract infections (over 90% of them caused by viral pathogens) in subjects taking probiotics and a small but significant reduction in disease severity in those infected^39^. The possible mechanisms involved in the probiotic protection against viruses are increased levels of type I interferons, the number and activity of antigen-presenting cells, NK cells, T cells, and the levels of systemic and mucosal specific antibodies in the lungs^40^ ^,^ ^41^. Probiotics are administered by the oral route, and their primary target is gut microbiota, which is also disrupted in SARS-CoV-2 infection^42^. However, the intense crosstalk between intestinal and pulmonary microbiotas mediated by microorganisms, immune cells, and their products benefit both microbiotas^43^. 

### The 4 ^th^ barrier: Antimicrobial peptides - defensins and lactoferrin 

Defensins are antimicrobial peptides produced by neutrophils (α-defensin) and epithelial cells (β-defensins). They are induced by microbial products or pro-inflammatory cytokines and exert multiple effects against viruses, including direct antivirus activity, modification of viral pathogenesis, and modulation of antiviral immune responses^44^ ^,^ ^45^. Defensins are potent chemotactic agents that induce migration of innate and adaptive immunity cells^46^, promote phagocytosis and present anti-inflammatory activity^47^. Their immunomodulatory effects include activating immune cells and modulating cytokines’ expression and secretion^44^ ^,^ ^48^. In addition, defensins display direct antiviral activity on respiratory viruses^49^ by targeting viral envelopes, glycoproteins, capsids, inhibiting viral fusion, and providing post-entry neutralization^50^. 

Studies on the effect of defensins on coronaviruses are still limited. Recent in silico^51^ and in vitro data^52^ showed that human β-defensin-5 could block the binding of SARS-CoV-2 to ACE2+ cells. An in vivo study showed that the prophylactic treatment of BALB/c mice with theta-defensin-1 from rhesus monkey before the infection with a mouse-adapted strain of SARS-CoV-1 caused 100% survival, in contrast to 75% of untreated mice, with a modest reduction in lung pathology and without a reduction in virus titer^53^. Moreover, Zhao and colleagues showed that a short peptide derived from mouse β-defensin-4 exhibited potent antiviral activity to SARS-CoV and MERS-CoV and different serotypes of influenza A virus^54^. The authors have demonstrated that the defensin peptide bound to viral particles enters the cells via endocytosis and prevents the endosomal acidification, which blocked the membrane fusion and subsequent viral RNA release^54^. That finding opens an avenue for developing new prophylactic and therapeutic agents with broad-spectrum antiviral activities, such as exploring human defensins as vaccine adjuvants^55^. The demonstration that vitamin D induces the synthesis of β-defensins by the respiratory tract’s epithelial cells^56^ poses the possibility that its deficiency might influence the prognosis of Covid-19 patients. Indeed, an association between vitamin D deficiency and the severity/mortality of Covid-19 has been demonstrated^57^ ^,^ ^58^, highlighting its possible use for improving defensin status^57^ ^,^ ^59^.

Another antimicrobial peptide acting on the respiratory tract’s antiviral defense is lactoferrin, considered the most versatile molecule of the organism due to the multitude of functions it exerts^60^. Lactoferrin is a glycoprotein synthesized by mucosal epithelial cells and neutrophils, and its multiple activities rely on its ability to sequestrate metals, especially iron^60^. The powerful immunomodulatory effects lactoferrin exerts arise from its ability to strongly bind to negatively charged structures, including immune cells receptors (e.g., toll-like, cytokine, and chemokine), DNA and RNA molecules (from host cells, viruses, and other infectious agents), as well as to microbial immunomodulatory molecules, such as bacterial lipopolysaccharide^60^ ^,^ ^61^. Lactoferrin acts on both innate and adaptive immune cells. It modulates the production of pro-inflammatory cytokines and type I interferons by macrophages and enhances their phagocytic activity; influences the maturation, migration, antigen uptake, and presentation by dendritic cells*;* suppresses the release of extracellular traps by neutrophils; stimulates cytotoxicity and production of IL-18 and type I interferon by NK cells; modulates T lymphocyte maturation, differentiation, activation, and the balance between Th1 and Th2 subsets; and promotes B lymphocyte maturation, enhances their capacity of antigen presentation, and secretion of IgA and IgG^60^ ^,^ ^62^ ^,^ ^63^. 

Lactoferrin exerts a broad range of antiviral activity on both RNA and DNA viruses due to its ability to strongly bind to their negatively charged components (surface epitopes, nucleic acids) and host cells (membrane receptors, nucleic acids), therefore inhibiting cell invasion and intracellular replication^64^ ^,^ ^65^. The antiviral effect of lactoferrin on respiratory viruses has been well documented^66^. It has been found that lactoferrin exerts its function in SARS-CoV-1 infection by enhancing NK cell activity and stimulating neutrophil aggregation and adhesion^67^. An in vitro study demonstrated that lactoferrin binds to heparan sulfate proteoglycans at the host cell surface, blocks the preliminary interactions of SARS-CoV pseudovirus with the host cell, and inhibits its entry into the cell^68^. These findings have encouraged the proposition of using exogenous lactoferrin to prevent and treat SARS-CoV-2 infection^69^ ^,^ ^70^. 

### The 5 ^th^ barrier: Secretory IgA antibodies 

SARS-CoV-2 virions must overcome another barrier to promote productive infection: the secretory IgA antibodies. Besides possessing a protective secretory component attached to their dimeric molecule, secretory IgA displays polyreactivity. It binds to numerous microbial antigens with low affinity, including lipopolysaccharides, DNA, flagellin, capsular polysaccharides, and virus components^71^ ^,^ ^72^. The polyreactivity of secretory IgA antibodies allows these molecules to play an essential role in the protection and homeostatic regulation of mucosal surfaces by separating the pathogen-laden outside environment from the inside of the body. 

The secretory IgA barrier exerts two protective functions: it facilitates the beneficial local microbiota’s permanence and helps to eliminate noxious agents, as pathogens and pollutant particles. The finding that a substantial fraction of the microbiota components is coated with IgA antibodies without any detrimental effect suggests its functional and evolutionary relevance. Indeed, experimental data indicate that polyreactive IgA antibodies bind to microbial structures and facilitate the clustering of pathogens at the mucus layer, securing the niche from invasion by competing species^71^ ^,^ ^73^. Furthermore, secretory IgA molecules contribute to the survival and diversity of microbiota^74^. 

The antimicrobial effects of IgA antibodies occur at three different sites of the mucosa: (1) at the lamina propria - IgA can neutralize invading pathogens that have penetrated through breaches in the inflamed epithelium and are subsequently cleared as immune complexes; (2) during transcytosis - the crossing of the epithelial cell by IgA from the lamina propria, where it is produced, to the surface of the mucosa, by intercepting occasional incoming pathogens that are further eliminated; (3) at the surface of the mucosa - by neutralizing, delaying or abolishing the invasion of the epithelium by pathogens^75^ ^,^ ^76^. Secretory IgA antibodies can restrain viruses at each of these three mucosa sites^77^ ^,^ ^78^, and their polyreactivity can provide cross-protection against infections with different strains and probably other species of viruses^79^ ^,^ ^80^. However, although both SARS-CoV-2 infection^81^ and vaccination^82^ induce secretory IgA antibodies, their role in protection remains unsettled. 

Since the microbiota induces secretory IgA, it is crucial to avoid its disruption by drugs (antibiotics, corticosteroids) or smoking, while probiotics^39^ can strengthen this defense barrier. Moreover, exogenous lactoferrin can enhance IgA synthesis^83^, especially if associated with retinoic acid^84^. In addition, the synthesis of secretory IgA could be stimulated by mucosal vaccines targeting SARS-CoV-2 given by oral or nasal routes^85^. 

### The 6 ^th^ barrier: Respiratory epithelial cells 

For coronaviruses and eight other human respiratory tract viruses, the respiratory epithelium is, at the same time, the target of the infection and a barrier against it. In the epithelial cell’s interior and using its resources, viruses replicate and subsequently shed virions to invade other cells. However, the respiratory epithelial cells display various mechanisms to counter virus invasion and help keep the organism’s homeostatic equilibrium. Firstly, it functions as a physical barrier against invaders. Respiratory epithelial cells, which cover the whole mucosal surface in contact with the air, are tightly attached, forming an effective mechanical barrier to the virus entry and dissemination into the submucosa. Furthermore, they hinder viral access to receptors within the basolateral epithelial membrane, which is a significant entry site for several viruses^86^ ^,^ ^87^. As argued previously, the respiratory epithelium’s function as a physical barrier also includes the mucociliary escalator and mucin production that form the mucus layer. 

The second mechanism used by respiratory epithelial cells to counter virus infection is producing antiviral molecules (interferons, lactoferrin, and defensins) that activate innate and adaptive antiviral immunity. Interferons exhibit both a direct antiviral effect and an indirect one by acting on immune cells. Type I interferons (IFN-α and IFN-β) bind to ubiquitously expressed cell receptors and induce the expression of hundreds of genes, which serve to limit further virus spread and infection. The direct antiviral activity of type I interferons includes impairment of the viral processes of cell entry, replication, transcription, translation, and the degradation of viral nucleic acids and proteins. Type I interferons also act on the immune cells by causing enhancement of phagocytosis, maturation of dendritic cells, and stimulation of cytokines and chemokines production by respiratory epithelial cells^88^. Type III interferons (IFN-λ1, IFN-λ2, and IFN-λ3) use a distinct receptor complex for signaling. They are expressed on only a few cell types, including respiratory and gastrointestinal epithelial cells, and trigger highly similar gene expression as type I interferons, suggesting that both IFN types might serve similar functions^89^. 

Notwithstanding the variety of sophisticated antiviral mechanisms displayed by the respiratory epithelial cells and the fact that SARS-CoV-2 is sensitive to interferons produced by these cells^90^ ^,^ ^91^, this virus exhibits a worrying ability to overcome this barrier by disrupting the tight junction formation that maintains the integrity of the epithelium^92^. Moreover, it has been found that SARS-CoV-2 causes cell fusion, apoptosis, destruction of epithelium integrity, cilium shrinking, and beaded changes in human airway epithelium cultures^93^. In addition, it has been suggested that interferons play a role in disrupting the epithelial cell barrier during SARS-CoV-2 infection^94^ and in its repair during recovery^95^. 

Smoking is a major disruptor of the epithelial cell barrier^96^, besides increasing the expression of SARS-CoV-2 receptor ACE2 in the respiratory tract epithelium^9^, and when in association with the virus, reduces interferon β-1 antiviral response and alters the stem cell-derived airway repair response^97^. Hence, avoiding smoking is crucial for maintaining the integrity of the epithelial cell barrier. On the other hand, zinc possibly plays a role in protecting the respiratory epithelium due to its antioxidant, anti-inflammatory, and anti-apoptotic effects. Furthermore, its ability to stabilize organelles acts as a cofactor for DNA synthesis and enhances wound repair^98^. 

### The 7 ^th^ barrier: Innate immunity 

The last constitutive respiratory defense barrier, the innate immunity, functions in close association with the sixth barrier, the epithelial cells, both maintaining productive crosstalk between them to fine-tuning their responses. The mechanisms of innate immunity are brought into play at the portal of entry of SARS-CoV-2, frequently the nasal goblet cells and ciliated cells of nose mucosa, which express both the ACE2 receptor and the protease TMPRSS2 necessary for host cell invasion. Interestingly, many of the top genes associated with the ACE2 gene code for innate immunity functions with antiviral activity^99^. Different cell types (monocytes, macrophages, dendritic cells, innate lymphoid cells, granulocytes, and epithelial cells) and molecules (complement, surfactant, mannose-binding lectin, cytokines, and chemokines) participate in the mechanisms of innate immunity^100^ ^-^ ^102^. The sensing of virus molecules by the cells of innate immunity elicits a cascade of intra- and intercellular signals with the potential to inhibiting the development of productive infection, thereby preventing or at least mitigating illness before adaptive immunity is activated. As a countermeasure against the elaborate human defense mechanisms, SARS-CoVs develop ways to circumvent or suppress the innate immune responses to ensure a window of opportunity for efficient replication, eventually followed by disease^103^. 

Macrophages are the most important cells of the innate immune system at the portals of entry of SARS-CoV-2. They play significant roles in detecting viruses and virus-infected cells, clearing apoptotic/damaged cells, and inducing and regulating adaptive immune responses. Alveolar macrophages abundantly secrete cytokines, chemokines, and growth factors that ensure rapid and effective communication with epithelial, stromal, dendritic cells, T regulatory lymphocytes, and innate lymphoid cells in the pulmonary environment^104^ ^-^ ^106^. They, therefore, act as a coordinator of local antivirus response. The demonstration that the viral load of SARS-CoV-2 peaked during the first week of illness then gradually declined over the second week^107^ ^-^ ^110^, which means before the full development of the adaptive immune response, points to the importance of the innate immune system for controlling SARS-CoV-2 infection. The existence of individuals showing RT-PCR positive tests for the virus who, a few days later, became negative without showing any symptom attributable to Covid-19^111^ ^,^ ^112^ is also a possible indication of the efficiency of the innate immunity to overcome the infection. Moreover, innate immunity mechanisms can control SARS-CoV-1 infection of mice in the absence of CD4+ and CD8+ T lymphocytes and antibodies^113^. 

Since the excessive activation of macrophages may lead to hyperinflammation, a significant cause of disease severity and death in SARS-CoV-2 infection^114^, regulating their function may strengthen the innate immunity barrier of defense. Different compounds seem to display the ability to modulate macrophage functions, including zinc^115^, vitamin D^116^, thalidomide^117^, probiotics^118^, omega-3 fatty acids^119^, metformin^120^, curcumin^121^, and coenzyme Q10^122^. Therefore, the investigation of their possible use to mitigate Covid-19 severity is warranted. 

## CONCLUSION

Before causing a productive infection, SARS-CoV-2 must overcome seven constitutive respiratory defense barriers and an elicited one, the adaptive immunity (discussed elsewhere^123^). The fact that months after its emergence, SARS-CoV-2 had infected over 100 million people indicates that the virus possesses a piece of machinery that allows it to evade all those defense barriers. Some of these mechanisms include the impairment of interferon production by host cells, the ability to hide immunogenic motifs from cell receptors, the concealment of viral RNA to avoid detection by cell sensors, the triggering of human defense cell death, and the impairment of lymphocyte functionality. This remarkable ability to evade human defense mechanisms implies that precursors SARS-CoV-2 of have probably been circulating among humans for a reasonable yet unknown time before the pandemics started. During this adaptation phase, virus variants were gradually ‘learning’ how to overcome the different human defense barriers until a full-brown variant emerged in December 2019, leading to the Covid-19 pandemic. It is anticipated that as far as adaptation progresses under the selective pressure of the immune system, the infection’s destructive burden will reduce, and eventually, SARS-CoV-2 may become part of human respiratory virome, as had occurred with other coronaviruses.
